# To the Origin of Fungi: Analysis of MFS Transporters of First Assembled *Aphelidium* Genome Highlights Dissimilarity of Osmotrophic Abilities between Aphelida and Fungi

**DOI:** 10.3390/jof9101021

**Published:** 2023-10-16

**Authors:** Igor R. Pozdnyakov, Evgeniy V. Potapenko, Elena S. Nassonova, Vladislav V. Babenko, Daria I. Boldyreva, Victoria S. Tcvetkova, Sergey A. Karpov

**Affiliations:** 1 Zoological Institute, Russian Academy of Sciences, St. Petersburg 199034, Russia; sakarpov4@gmail.com; 2 Institute of Evolution, University of Haifa, Haifa 3498838, Israel; potapgene@gmail.com; 3 Department of Evolutionary and Environmental Biology, University of Haifa, Haifa 3498838, Israel; 4 Laboratory of Cytology of Unicellular Organisms, Institute of Cytology, Russian Academy of Sciences, St. Petersburg 194064, Russia; nosema@mail.ru; 5 Department of Invertebrate Zoology, Faculty of Biology, St. Petersburg University, St. Petersburg 199034, Russiacvetkoviktoriya@yandex.ru (V.S.T.); 6 Lopukhin Federal Research and Clinical Center of Physical-Chemical Medicine, Federal Medical Biological Agency, Moscow 119435, Russia; daniorerio34@gmail.com (V.V.B.);

**Keywords:** Aphelida, fungi, Holomycota, osmotrophy, MFS proteins, evolution, genome, sugar porters

## Abstract

Aphelids are a holomycotan group, represented exclusively by parasitoids infecting algae. They form a sister lineage to Fungi in the phylogenetic tree and represent a key group for reconstruction of the evolution of Holomycota and for analysis of the origin of Fungi. The newly assembled genome of *Aphelidium insullamus* (Holomycota, Aphelida) with a total length of 18.9 Mb, 7820 protein-coding genes and a GC percentage of 52.05% was obtained by a hybrid assembly based on Oxford Nanopore long reads and Illumina paired reads. In order to trace the origin and the evolution of fungal osmotrophy and its presence or absence in Aphelida, we analyzed the set of main fungal transmembrane transporters, which are proteins of the Major Facilitator superfamily (MFS), in the predicted aphelid proteomes. This search has shown an absence of a specific fungal protein family Drug:H^+^ antiporters-2 (DAH-2) and specific fungal orthologs of the sugar porters (SP) family, and the presence of common opisthokont’s orthologs of the SP family in four aphelid genomes. The repertoire of SP orthologs in aphelids turned out to be less diverse than in free-living opisthokonts, and one of the most limited among opisthokonts. We argue that aphelids do not show signs of similarity with fungi in terms of their osmotrophic abilities, despite the sister relationships of these groups. Moreover, the osmotrophic abilities of aphelids appear to be reduced in comparison with free-living unicellular opisthokonts. Therefore, we assume that the evolution of fungi-specific traits began after the separation of fungal and aphelid lineages, and there are no essential reasons to consider aphelids as a prototype of the fungal ancestor.

## 1. Introduction

Aphelida is a group of algal parasitoids represented by an intracellular ameboid-plasmodial trophic stage and exiting to the environment zoospores that infect other algal cells [1,2,3]. Phylum Aphelida belongs to Holomycota, one of two branches of Opisthokonta [4,5] and, as shown by phylogenomic analysis, is a sister group to Fungi, very distinctive and specialized osmotrophic organisms [6,7,8,9].

After the exclusion of the fungi-like stramenopiles, Fungi became a distinct monophyletic taxon within Opisthokonta, uniting osmotrophic organisms with mycelial or pseudomycelial organization and a chitinous cell wall [4,10,11,12]. Unambiguous characterization of this taxon is difficult because of secondary changes in some of its representatives [13]. Conventionally, “higher”, or crown, fungi are completely devoid of flagella, while the “lower”, or basal, fungi have a flagellated stage in their life cycle, the zoospores [4,10]. The sister relationship of Aphelida and Fungi seems surprising because the representatives of the former group, being amoeboid and phagotrophic, have only superficial similarities in the life cycle with the members of the latter one [2]. Meanwhile, aphelids are closer to fungi than to *Rozella* spp., which are similar to algal parasitoids both in life cycle, cell morphology and phagotrophic mode of feeding [5,6,9].

The fact that fungi are related to such different organisms gives a possibility to identify early stages of fungal specialization since it remains unclear how the fungal features originated and evolved. Obviously, the fungal traits could not arise simultaneously, but had to increase gradually until they reached a pronounced morphological appearance. Therefore, it is possible that the organisms related to fungi could retain some fungal features in an ancient, inchoate, or not fully formed state, having inherited them from a common ancestor with fungi.

In the field of taxonomy, such searches and finds can clarify the frames of the Fungi, since the problem of apomorphies and borders of this taxon has not yet been resolved. Some “classic” fungal signs, such as osmotrophy, are not only characteristic of Fungi. Other features, such as mycelial growth and chitin cell walls, are repeatedly lost in undoubted representatives of fungal lineages [13]. The idea of fungal apomorphies became even more uncertain after the inclusion of phagotrophic aphelids, rozellids, and microsporidia in Fungi [7]. The most recent and comprehensive review of eukaryotic taxonomy states: “There are no unambiguous morphological, subcellular, or biochemical synapomorphies of fungi” [4]. The detection or non-detection of any latent features of fungi in closely related organisms may be an additional argument pro or contra their inclusion in the Fungi.

We suggested that aphelids can have increased osmotrophic capabilities compared to free-living opisthokonts, despite the fact that they retain phagotrophic nutrition. We had several reasons for this assumption. (1) A common ancestor of aphelids and fungi could already have a heightened capacity for osmotrophy, which could have been inherited by the aphelids. (2) For aphelids, the increase in osmotrophy may be adaptive, since their trophonts are immersed in the host cytoplasm, which is rich in nutrient molecules. (3) The repertoire of proteins associated with digestive vacuoles, especially the COMMD/CCDC22/CCDC93 (CCC) complex, is somewhat reduced in aphelids [9]. This reduction may be a synapomorphy of both, the aphelids and fungi, inherited from a common ancestor. Such a reduction can be justified precisely in the case when the osmotrophic mechanism of nutrition was strengthened in the common ancestor. To verify this assumption, we searched for Major Facilitator superfamily (MFS) proteins, which are a key component of osmotrophic machinery, in the predicted aphelid proteomes, and carried out their comparative analysis.

The MFS superfamily is a large group of plasma membrane proteins that are present in all cellular organisms and serve as transmembrane transporters of various substances, including mono- and oligosaccharides, metabolites, amino acids and oxyanions [14,15]. The main feature of MFS transporters is the presence of usually 12 (sometimes 14) transmembrane helices, which are divided into two 6-helix blocks opposite each other and connected by an extended loop. This molecular composition is often referred to as the MFS domain [15,16,17]. The MFS superfamily is divided into different families, whose proteins differ in their mechanism of operation, transported substrates and features of function [15,16,18].

MFS transporters in fungi are numerous and diverse because fungi, as obligate osmotrophic organisms, must transport all types of nutrient substrates across the membrane [19,20,21]. MFS proteins in fungi are represented both by families common to all organisms, such as Sugar Porters (SPs), and by fungal-specific families, such as Drug:H^+^ Antiporters-2 (DHA-2) [18,19,22,23,24]. Multiple and specialized SP proteins perform a function of transport of various carbohydrate substrates. DHA-2 proteins carry various substances, including nitrogen-containing ones. Thus, the proteins of these two MSF families play a major role in fungal nutrition.

In this article, we present a de novo assembled genome of *Aphelidium insulamus*. Based on the predicted proteomes derived from the available aphelid genomes and transcriptomes, we searched for SP and DHA-2 proteins and compared them qualitatively and quantitatively with corresponding proteins of dikaryan and zoosporic fungi, the unicellular parasitoid holomycotan *Rozella allomycis* and representatives of Holozoa.

## 2. Materials and Methods

### 2.1. DNA Extraction and Genome Sequencing

The DNA was extracted from the strain X-133 of *Aphelidium insulamus* maintained in the culture collection of parasitic protists (CCPPs) of Zoological Institute Russian Academy of Sciences (ZIN RAS) [25] using two different protocols. In the frames of the first protocol, DNA was purified from the heavily infected culture of *Tribonema gayanum*, containing almost digested algal cells with trophonts and plasmodia of *A. insulamus*. DNA was extracted using a Wizard^®^ Genomic DNA Purification Kit (Promega, Madison, WI, USA) according to the manufacturer’s protocol. Purified DNA was used for genome sequencing on the Oxford Nanopore platform.

According to the second protocol, DNA was extracted from zoospores, which were isolated from the infected algal culture and concentrated by centrifugation in an Eppendorf MicroSpin centrifuge at 4300× *g* for 10 min. Sedimented cells were used for the Multiple Displacement Amplification (MDA) with Repli-g Single Cell Amplification Kit (Qiagen, Venlo, The Netherlands), according to the manufacturer’s protocol for single-cell reactions. To avoid biases of uneven whole genome amplification, ten independent MDA reactions were performed, each yielded ca 5.5–8.0 µg DNA. The MDA products were checked for the presence of target DNA using PCR amplification of a fragment of the SSU rRNA gene with universal eukaryotic primers S12.2 and RibB [26]. PCR amplification program: 5 min denaturation at 94 °C, 35 cycles of a denaturation step at 94 °C for 15 s, a 30 s annealing step at 50 °C and an extension step at 72 °C for 2 min, and a final elongation step of 7 min at 72 °C. The positive DNA samples were mixed in equimolar proportion and used for library preparations and sequencing on Illumina HiSeq4000 and Oxford Nanopore platforms.

For Illumina HiSeq4000 sequencing two paired-end libraries were prepared following the TruSeq and Nextera library preparation protocols with an insert length of 700 bp. A total of 62 million and 83 million paired-end reads were obtained for the two libraries. 

The long reads were generated with MinION and PromethION sequencing (Oxford Nanopore Technologies, Oxford, UK). The sequencing libraries were prepared using the ligation sequencing kit SQK-LSK109, native barcoding expansion kit EXP-NBD104 and EXP-NBD114. The gained library for ONT sequencing was then loaded into the flow cells (FLO-MIN106 and FLO-PRO002). Thus, two libraries were obtained with 560,000 and 300,000 long reads.

### 2.2. Genome Assembly and Annotation

The initial genomic assemblies were performed with Flye, v.2.9.1 [27] with default settings using two libraries. The draft assembly was checked for contaminations with BlobToolKit v2.3.3 [28]. Trusted contigs were selected based on the annotation of contigs against NCBI nucleotide and UniProt reference proteomes databases, GC content and short/long reads coverage information. Further, both long reads libraries were mapped on the trusted contigs using the Minimap 2, v.2.24 [29] and all unmapped reads were discarded. The next step assembly was made with the trusted reads with Flye, v.2.9.1 using the same way. The new assembly was polished using the Illumina paired read libraries. The first step of polishing was made in Racon, v.1.4.3 [30] and the second in Pilon, v.1.24 [31]. The assembly quality was controlled with Busco, v.5.4.2 [32] and QUAST, v.5.0.2 [33] on every step of assembly.

Structure annotation of assembled genome was carried out using funannotate pipeline, v.1.8.13 [34], which includes repeat masking with tantan, ab initio gene-prediction training (Augustus, PASA, SNAP, GlimmerHMM, GeneMark), generating consensus gene model (Evidence Modeler) [35] and functional annotating of proteins against several databases (Pfam, InterPro, GO, dbCAN, BUSCO, MEROPS, EggNog, COG). We improved gene prediction with protein evidence from UniProt database and transcript evidence datasets of closely related species: *Aphelidium insulamus* X-134_O14 [25] and *A. tribonematis* X-102_P2.

Secreted proteins were estimated with the Phobius web service [36] and tRNAs were predicted in silico with a tRNAscan-SE algorithm [37] included in the funannotate pipeline. Structure and functional comparison with genomes of related species (*Amoeboaphelidium protococcarum*, *A. occidentale*) and zoosporic fungi (*Gonapodya prolifera*, *Blyttiomyces helicus*, *Powellomyces hirtus*, *Spizellomyces punctatus*) were performed with the compare command of the funannotate pipeline. All considered genomes before analysis were re-annotated with InterProScan [38] on the same database versions.

For functional comparison, all analyzed genomes were (re)annotated with InterProScan. To estimate PFAM motif occurrence in holomycotan genomes, we applied non-metric multidimensional scaling (NMDS) projection of a Bray–Curtis distance matrix implemented in the compare command of the funannotate pipeline.

### 2.3. The Selection and Analysis of MFS-Domain Proteins

**Figure 1:**
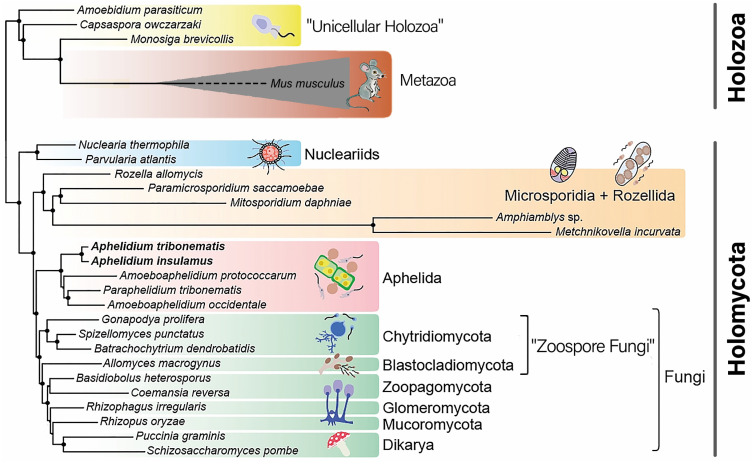
Phylogenetic tree of the Opisthokonta supergroup. After: Galindo et al. (2022) [8], modified. Non-taxonomic paraphyletic groups are written in quotation marks.

Phylum Aphelida, and a solid clade of zoosporic and filamentous fungi, belong to Holomycota. The latter together with Holozoa forms the supergroup Opisthokonta (Figure 1). Our key objective was to highlight MPS proteins demonstrating symplesiomorphic characteristics present across all Opisthokonta, synapomorphic characteristics exclusive to aphelids and fungi, and, if they exist, autapomorphic characteristics unique to aphelids and fungi individually. Therefore, we focused only on two specific families of MPS proteins (SP and DHA-2). Consequently, the challenge was to meticulously select search parameters to ensure robust identification of these family-specific proteins in representatives of the Opisthokonta.

The MFS-domain proteins were initially chosen by the BLAST searching in the predicted proteomes of selected organisms with annotated genomes. For the search, the web interface of NCBI BLASTP [39] was used. To select search parameters, we first selected well-annotated genomic assemblies of representatives of Opisthokonta: fungi, sister group to Aphelida and Metazoa, the largest taxon in the Holozoa, which is sister to Holomycota (Figure 1). The criteria of selection were: (1) free-living lifestyle, the organism should not be an obligate parasite with very divergent food specializations; (2) the number of predicted proteins in the assembly; (3) completeness of functional annotation for calibrating search parameters based on proteins with known membership and function. As a result, the genome assemblies (NCBI genome ID in brackets) of dikaryan fungi *Saccharomyces cerevisiae* (559292), *Neurospora crassa* (367110), *Cryptococcus neoformans var. neoformans* (214684), zoosporic fungi *Gonapodya prolifera* (1344416) (Chytridiomycota), *Spizellomyces punctatus* (645134) (Chytridiomycota), early diverging metazoans *Amphimedon queenslandica* (2698) (Porifera) and *Stylophora pistillata* (18227) (Cnidaria) were chosen.

The *S. cerevisiae* glucose sensor NP_010087.1 was taken as the initial query for the search for SP proteins. The proteins of DAH-2 family were searched in the aforementioned fungal genomes with the initial query NP_011740.3, which is a *S. cerevisiae* azole transporter. The E-value and bit-score thresholds were set to 1 × 10^−5^ and 50, correspondently.

Among the BLASTP hits, the proteins of desired families were selected based on annotations and the found sequences were downloaded as two databases for SP proteins and DAH-2 proteins separately. The hidden Markov models were built by the hmmbuild program of the hmmer, v.3.3.2 batch [40] for SP and DAH-2 proteins separately. 

The test search using the hmmsearch program was performed against the proteins of an extended set of organisms: additional representatives of Opisthokonta, distantly related green plant *Arabidopsis thaliana* and some prokaryotic organisms. *Blyttiomyces helicus* was included in the search, despite the low quality of the genome assembly, as a representative of a divergent lineage within Chytridiomycota with an unclear position. An expectation was held that such a genome might present specific MFS proteins.

As a result, the search among the predicted proteomes described above and additional proteomes of *Agaricus bisporus* var. *bisporus* (936046) (Fungi, Dikarya), *Blyttiomyces helicus* (388810) (Fungi, Chytridiomycota), *Powellomyces hirtus* (109895) (Fungi, Chytridiomycota), *Salpingoeca rosetta* (946362) (Holozoa, Choanoflagellata), *Arabidopsis thaliana* (3702) (Viridiplantae, Embryophyta), *Escherichia coli* (167) (Bacteria; Gammaproteobacteria), *Acetilactobacillus jinshanensis* (1720083) (Bacteria, Bacilli) showed that bitscore threshold 250 allows to find the proteins of desired families and cut off the proteins of the other families, even related ones.

For the final search, we selected well-annotated genomic assemblies from representatives of various subgroups of Opisthokonta. At this step, the SP and DAH-2 proteins were chosen from the predicted proteomes of *Amoeboaphelidium protococcarum* (114058) (Holomycota, Aphelida), *A. occidentale* (114059), *Paraphelidium tribonematis* and *Aphelidium insulamus*, as well as the predicted proteomes of *Agaricus bisporus* var. *bisporus* (936046) (Fungi, Dikarya), *Blyttiomyces helicus* (388810) (Fungi, Chytridiomycota), *Powellomyces hirtus* (109895) (Fungi, Chytridiomycota), *Synchytrium endobioticum* (286115) (Fungi, Chytridiomycota), *Salpingoeca rosetta* (946362) (Holozoa, Choanoflagellata), *Monosiga brevicollis* (431895) (Holozoa, Choanoflagellata), *Capsaspora owczarzaki* (595528) (Holozoa, Filasterea) and *Rozella allomycis* (12422) (Holomycoa, Rozellida) by the searching of correspondent profiles against these proteomes using hmmsearch. The predicted proteome of *P. tribonematis* is absent in the NCBI databases and it was downloaded from the Figshare store [6]. The predicted proteome of *A. insulamus* was obtained using the genome sequenced and assembled in the frames of the present study and the pipeline described above.

The phylogenetic analysis was performed for the found sequences of sugar transporters. The multiple sequence alignment (MSA) was prepared in the M-Coffee aligner using the web server interface (https://tcoffee.org, accessed on 17 November 2022) [41]. The MSA was treated in TrimAl, v.1.4.rev15 [42] with a gap threshold of 0.5 for the removal of columns with the gap abundances appeared due to the large divergence between protein sequences.

The initial tree was constructed on the ground of a trimmed MSA using IQ-Tree 2, v.2.0.3 [43] with the settings of automated determination of the substitution model and 1000 replicates of an ultrafast bootstrap. The most suitable substitution model was determined as LG+F+G4. For the final tree construction IQ-Tree 2, v.2.0.3, was also used, with LG+F+G4 substitution model and 100,000 replicates of an ultrafast bootstrap.

The sequences of bacterial SP proteins (*Bacteroidales bacterium*, *Acetilactobacillus jinshanensis*, *Bacteroidota bacterium*, *Lentilactobacillus* spp., *Secundilactobacillus hailunensis*) showing the best BLAST matches for some aphelid proteins were downloaded from the NCBI database by their accessions. A phylogenetic tree including aphelid, fungal and bacterial SP proteins was constructed as described above.

Prediction of transmembrane structures in SP proteins was performed using the web service TMHMM-2.0 (https://services.healthtech.dtu.dk/service.php?TMHMM-2.0, accessed on 17 November 2022).

Since in the case of *A. protococcarum*, the search showed the presence of eight separate SP proteins, which form two quartets on the phylogenetic tree, we specifically assessed the level of similarity of these proteins using reciprocal BLASTP alignments.

## 3. Results

### 3.1. Assembly of Aphelidium insulamus Genome

The hybrid assembly based on Oxford Nanopore and Illumina paired reads yielded an *A. insullamus* genome with a total length of 18,927,283 bp that were distributed among 274 scaffolds, with an N50 252,907 and a 52.05% GC percentage (NCBI BioProject accession number PRJNA902644). The assembled genome contains 5% of repetitive sequences. The average coverage of Nanopore long reads and Illumina paired reads were about 9.6-fold and 95.2-fold, respectively. We identified 7925 genes, including 7820 protein-coding ones. The average lengths of the predicted genes and proteins were 1664 nucleotides and 484 amino acids, respectively. Funannotate pipeline annotated the following number of predicted protein-coding sequences using different databases: InterPro (5893), Pfam (4957), GO (4439), EggNOG (3735), BUSCO Eukaryota Odb10 (287), MEROPS (268) and dbCAN (125). In addition, we annotated 1431 secreted proteins and 105 tRNA-encoding genes.

The comparison of the obtained assembly with the genome assemblies of two related aphelids and a selected set of zoosporic fungi in terms of key indicators showed its comparable characteristics (Table 1). The quality of assembly of *A. insulamus* is significantly inferior to only two assemblies: *Amoeboaphelidium protococcarum* and *Spizellomyces punctatus*. The quantitative indicators (assembly size, scaffolds number, N50, average and largest scaffolds, unique BUSCOs) of *A. insulamus* assembly are between the corresponding indicators of previously published genomes of *A. protococcarum* and *A. occidentale* [9]. 

Interestingly, the GC content of *A. insulamus* genome is more similar to fungi than to *Amoeboaphelidium* species. On the contrary, the number of proteins and BUSCO genes is closer to *A. occidentale*. 

**Figure 2:**
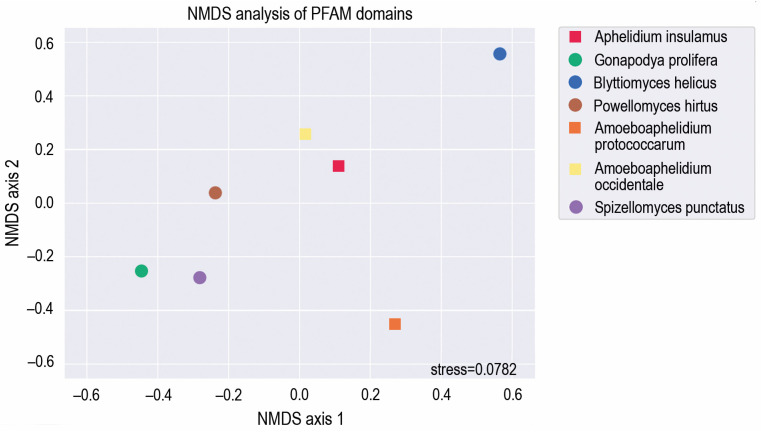
NMDS analysis showing PFAM domain co-occurrence in the genomes of *Aphelidium insulamus*, related aphelid species and zoosporic fungi. Fungal genomes are indicated by circles, and aphelid genomes are indicated by squares.

To arrange the species in a two-dimensional space based on the functionality of their genomes, we applied the Nonmetric Multidimensional Scaling (NMDS) algorithm to functional domain annotations, using abundance data of PFAM domains (Figure 2; Supplementary Table S1). Fungi (*Gonadopodya prolifera*, *Spizellomyces punctatus* and *Powellomyces hirtus*) were placed closer to each other than to aphelids, except the fungi *Blyttiomyces helicus.* We assume that the lack of clusterization of *B. helicus* with the rest of fungi is explained by the poor quality of its genome assembly. These results have showed that *A. insulamus* is very close to *A. occidentale* in the functional domain content, while *A. protococcarum* is rather distant from both of them, possibly due to the genome-wide duplications characteristic of the latter [9]. To check this hypothesis, we partitioned the count of PFAM domains in the genome of *A. protococcarum* by the count of corresponding domains within genomes of *A. insulamus* and *A. occidentale*. The median value of these distributions was two for both cases, indicating a twice higher occurrence of PFAM domains in the genome of *A. protococcarum* compared to the genomes of *A. insulamus* and *A. occidentale*. Such a ratio of domain likely underlies the species’ distant positioning from other aphelids, as shown in Figure 1. It is noteworthy that *P. hirtus* is placed approximately equidistant from fungi and aphelids, which may be attributed to the lower number of genes in *P. hirtus* (6536) in comparison to *G. prolifera* (13831) and *S. punctatus* (9422). In terms of overall domain composition, this positioning may bring *P. hirtus* closer to aphelids (*A. insulamus*—7822; *A. occidentale*—7495).

### 3.2. MFS Protein Analysis

Proteins of the DHA-2 family have not been found in any species of Aphelida, as well as in *R. allomycis*. A search for SP family proteins in aphelid genomes found only one SP protein in *P. tribonematis*, two SP proteins in *A. occidendale*, three proteins in *A. insullamus* and eight ones in *A. protococcarum* with a duplicated genome (see Discussion). The detected eight SP-porters of *A. protococcarum* are divided into two quartets of proteins with a very high level of identity (93–98%) within each, which shows their recent origin from two genes through a series of duplications.

The number of SP proteins comparable to that in aphelids was found in *C. owczarzaki* (1), *R. allomycis* (2) and *B. helices* (3). In all other studied opisthokonts, their number is higher (the case with *A. protococcarum* will be considered separately), e.g., Metazoa have more than a dozen of them and dikaryan fungi contain several dozens of SP proteins (Table 2). This is consistent with the diversity of fungal adaptations to various substrates and types of nutrition, as well as with the acquisition of nutrients through transmembrane transport from the internal fluids in most cells of multicellular animals.

**Figure 3:**
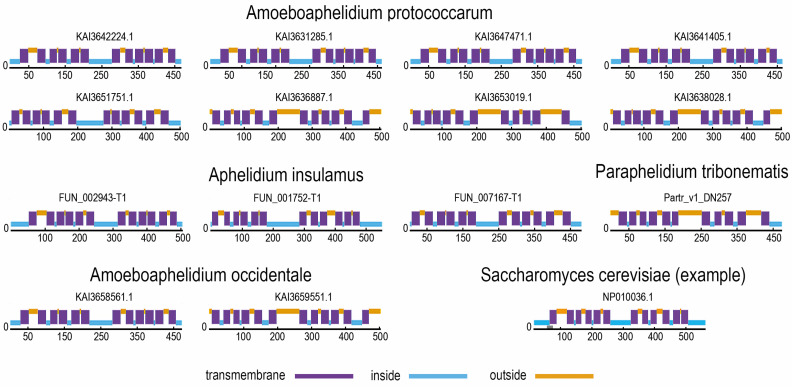
Prediction of distribution of intracellular, extracellular and transmembrane regions in the SP-protein molecule. The probable nature of the region is indicated by color (blue—intracellular, violet—transmembrane, orange—extracellular).

Prediction of the structures of aphelid SP proteins (Figure 3) shows that all of them have a typical structure with a canonical MSF domain with 12 transmembrane helices. This shows that the aphelid SP proteins have the correct structure for this protein family and, most likely, function normally.

**Figure 4:**
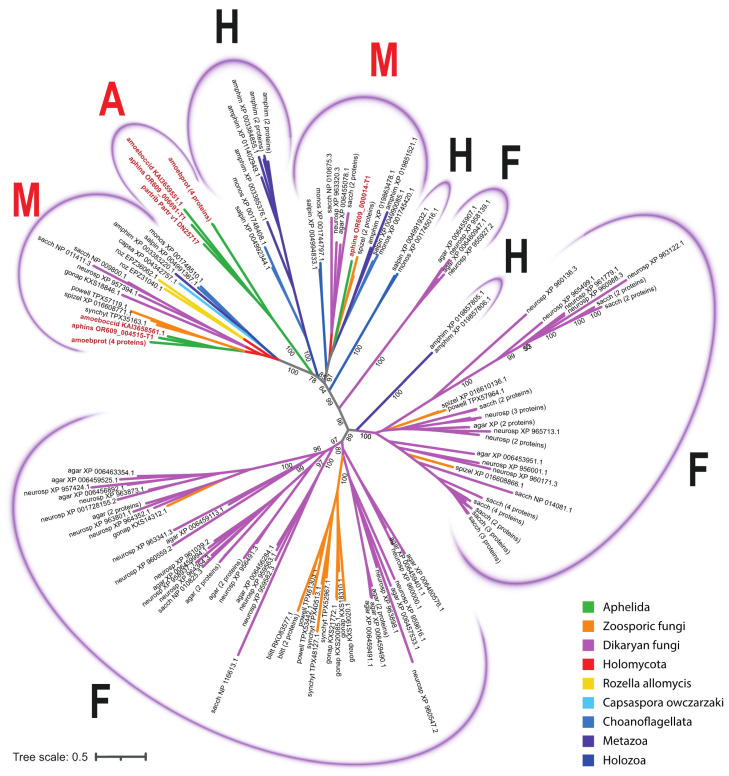
The phylogenetic tree of SP proteins of the studied opisthokont species. The opisthokont taxa are marked with colors. The label of leaf is the short designation of species (see Table 2) and the accession number of the sequence in NCBI. Aphelid sequences are labelled in red. The numbers indicate the support values (100,000 replicates of ultrafast bootstrap). A—aphelid clades, containing aphelid sequences only; F—fungal clades, containing sequences of zoosporic and dikaryan fungi only, H—holozoan clades, containing sequences of holozoan representatives; M—mixed clades, containing sequences of studied organisms both from Holomycota and from Holozoa.

**Figure 5:**
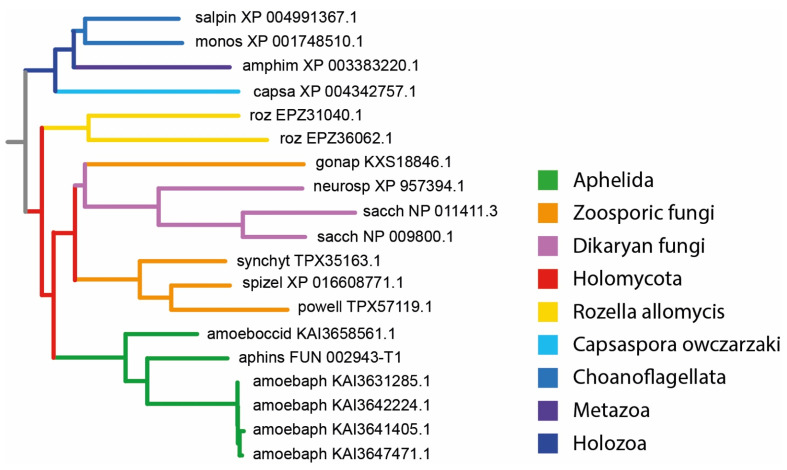
The branching order in the tree inferred from the analysis of orthogroup of SP proteins identified in the studied opisthokont species. Species designations used in the phylogenetic tree are provided in Table 2.

In the unrooted phylogenetic tree inferred from the analysis of aligned sequences of SP proteins, four variants of clades were observed: fungal, holozoan, aphelid and mixed; the latter ones include the orthologous proteins of opisthokonts from different groups (Figure 4; short version, the full version in Newick format is available in the Supplementary Tree S2). Three holozoan clades (Figure 4) include proteins of either choanoflagellates or choanoflagellates and the sponge *A. queenslandica*. Two mixed clades (Figure 4) include proteins of all the studied species: fungi, aphelids, *R. allomycis* and all holozoans. One (rarely two) proteins of each organism are present in these clades, except for *A. protococcarum*. The branching order observed within the mixed clades is consistent with recent multigene phylogenies [8,9]. Specifically, the aphelid proteins form a sister branch to the fungal proteins, while the proteins of *R. allomycis* represent a sister lineage to the group uniting the proteins of fungi and aphelids. In addition, the holozoan protein group is observed as a sister lineage to the group formed by holomycotan proteins. In the holozoan part of the clades, the proteins of *C. owczarzaki* branched off before the Choanoflagellata and Metazoa lineages (Figure 5). Three observed fungal clades (Figure 4) have many branching levels and contain many or at least several proteins of each fungal species grouped into internal clusters. Within them, specific subbranches of dikaryan fungi, zoosporic fungi and branches with proteins of both groups can be distinguished. The only aphelid clade (Figure 4) contains proteins of all aphelid species and does not contain proteins of other opisthokonts.

Thus, aphelid protein sequences fall into three clades: a specific one with aphelid sequences only (A), and two mixed ones with holomycotan and holozoan sequences (M). At the same time, the clades combining the protein sequences of aphelids and fungi without any holozoan sequences are absent.

**Figure 6:**
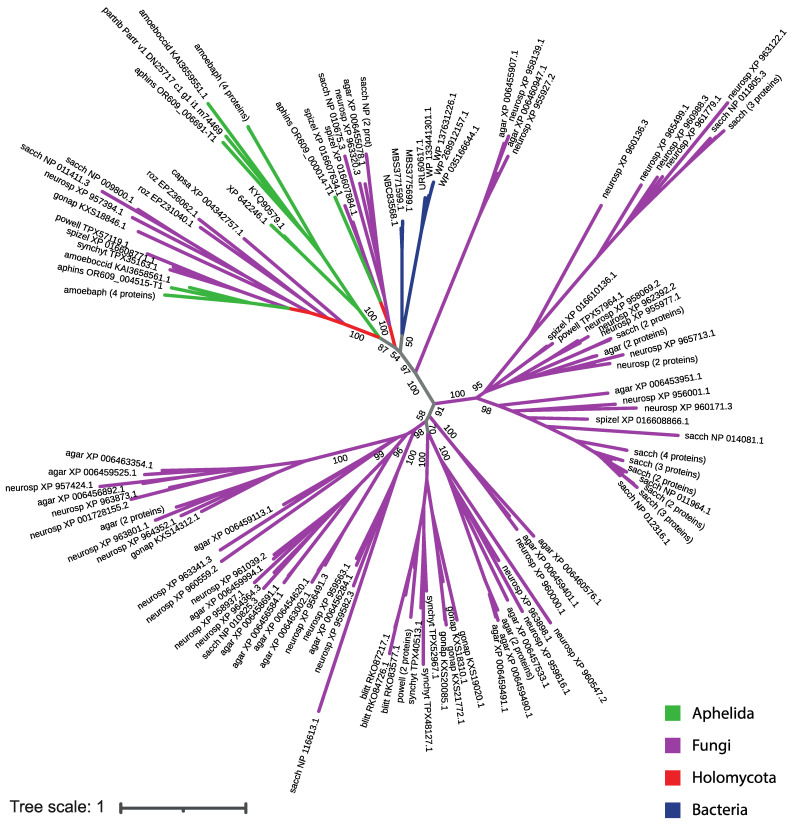
The phylogenetic tree of all found fungal and aphelid SP proteins together with the bacterial ones similar to SP proteins of the aphelid-specific clade. The opisthokont taxa are marked with color. The label of leaf is the short designation of species (see Table 2) and the accession number of the sequence in NCBI. The numbers indicate the support values (100,000 replicates of an ultrafast bootstrap).

Protein sequences from the aphelid-specific clade show the maximum similarity with SP sequences of bacteria and two proteins of dictyostelid amoebae in the BLAST search (Table 3), but do not show similarities with SP sequences of other opisthokonts, which could indicate the horizontal transfer of these genes from bacteria to aphelids and some amoebae. We performed a phylogenetic analysis of SP proteins found in fungi and aphelids. Bacterial SP proteins, which demonstrate the best match in BLAST with SP proteins of an exclusively aphelid clade, were also included in the analysis. It was shown that proteins of the aphelid-specific orthogroup cluster neither with bacterial proteins, nor with fungal and aphelid proteins (Figure 6; short version, the full version in Newick format is available in the Supplementary Tree S3). This observation suggests a complex and not yet understood evolutionary history of these proteins.

## 4. Discussion

Up to now the genomes of three strains of aphelids have been sequenced and annotated: *A. protococcarum* strains X5 and FD95 and *A. occidentale* [9]. The first assembled genome of a representative of the genus *Aphelidium* provided new data, which we used for a comparative study of MFS proteins in Opisthokonta. 

The species *A. insulamus* and *A. occidentale* exhibit a high degree of similarity, not only in terms of the core gene set and the number of protein-coding genes but also in terms of functionality, as evidenced by the similarity of their domain profiles. *A. protococcarum* is different from these two species. This observation correlates with a long distance between *A. occidentale* and *A. protococcarum* in the phylogenetic trees and with the demonstrated polyphyly of the genus *Amoeboaphelidium* [9].

Aphelid genomes contain SP protein genes belonging to three different orthogroups. *A. insulamus* contain the genes of all three orthogroups, showing maximum diversity. *A. protococcarum* and *A. occidentale* contains genes belonging to two different orthogroups. In *P. tribonematis*, only one sequence encoding the SP protein was found in the transcriptomic data. Eight SP genes of *A. protococcarum* are two quartets of very closely related paralogs that are the result of recent multiplications and belong to the same two orthogroups as both *A. occidentale* genes. This situation is consistent with the peculiar evolutionary history of *A. protococcarum* that underwent genome-wide duplications [9].

Two of three SP orthogroups including the genes of Aphelida also contain the orthologs of other opisthokonts, which are fungi, *R. allomycis* and Holozoa. Obviously, these orthologs are inherited from a common opisthokont ancestor. They retain a structure close to the ancestral one in all opisthokonts and have not undergone multiple duplications (except for *A. protococcarum*).

The third group of orthologs, containing only the SP genes of aphelids, can hypothetically originate from a gene obtained by an ancestor of aphelids from bacteria by horizontal gene transfer, but its origin has not yet been elucidated.

Specific clades of SP proteins, found in Metazoa, fungi and aphelids and absent in the common ancestor of opisthokonts, appeared in evolution probably after the separation of the corresponding lineages. We did not find orthogroups common to all Holomycota, but absent from Holozoa. This means that we do not see any orthogroups that could be lost in the Holozoa, or arose from a common ancestor of the Holomycota.

Specific orthogroups of fungi could hypothetically arise from their common ancestor with aphelids, and then be lost in aphelids. However, firstly, with an endobiotic lifestyle, the development and following strengthening of osmotrophic capabilities are usually observed, not their weakening. Secondly, the aphelids contain precisely those SP orthogroups found in all opisthokonts and have not even a single orthogroup specific for fungi. It seems that such a “neat” disappearance, especially in view of above-mentioned statement, is less likely than the appearance of specific orthogroups in fungi after their separation from the aphelids.

There are at least six reliable specific fungal clades on the constructed tree. Two of them are clearly divided into subclades, each containing proteins from different fungal taxa. Thus, there are about 6–11 specific fungal SP orthogroups. This fact agrees well with the tendency to the enhanced evolution of metabolic genes shown in fungi [20,44].

It is also clear that aphelids, as well as *R. allomycis*, have no fungi-specific SP proteins, similarly, neither aphelids nor *R. allomycis* have the fungi-specific DHA-2 family of MFS proteins.

The number of SP proteins in aphelids, *R. allomycis* and *C. owczarzaki* is minimal for opisthokonts. For *B. helicus* the lowest number of these proteins compared to other chytrids may be a result of incomplete genome assembly (see Table 1). In addition, the number and diversity of SP proteins in aphelids tend to decrease over the course of aphelid evolution.

All these facts indicate that aphelids do not show at the genomic level any signs of likeness to fungi in enhancing their osmotrophic abilities. Moreover, the osmotrophic abilities of aphelids even look reduced compared to free-living unicellular opisthokonts. One of the reasons could probably be a specialization to the endobiotic lifestyle. While it is advantageous for a free-living cell to have a wide set of trophic possibilities to cope with environmental challenges, an endobiont can have a more specialized feeding mode, since its environment is probably more stable. In the case of aphelids, feeding by phagocytosis turns out to be such a single option. Probably, the same reason may explain a limited repertoire of genes involved in the osmotrophy machinery observed in *R. allomycis* and *C. owczarzaki*.

These results are fully consistent with previously obtained data showing differences in the sets of receptor-like protein kinases and carbohydrate processing enzymes in aphelids and fungi [9]. All these differences suggest that the common ancestor of fungi and aphelids did not have any, even hidden, traces of fungal osmotrophy, which appeared in fungi after the separation of these two lineages.

Hence, ideas about the morphology and lifestyle of the common ancestor of aphelids and fungi become vaguer than just the assumption that it was aphelid-like. Recently, a number of common genes have been discovered in fungi and aphelids [6,8,9,20] and some metabolitic features of their common ancestor have been identified [8]. However, if we consider separately each of the common features of fungi and aphelids, they do not allow us to reconstruct a precise image of their common ancestor.

(1) The noted evidence that the common ancestor of fungi and aphelids fed on algae [8] does not indicate how such feeding occurred. If the cell walls of algae were the original substrate for fungi [45], then the path of transition from endobiotic cytoplasmophagy to extracellular digestion of algal cell walls remains unclear. Modern aphelids, although they have cellulases, do not use extracellular cleavage products and do not show the prerequisites for the formation of a fungal type of nutrition. 

In this regard, it is interesting that the possibility of extracellular degradation of polysaccharides was noted in recently discovered organisms from basal lineages of Holozoa [46]. Being cytoplasmophagous predators, these organisms are also capable of bacteriophagy, feeding on particles of solid carbohydrates, and extracellular degradation of carbohydrates. Possibly, the ancestral forms of opisthokonts could also have had a whole range of trophic possibilities, which were further developed in various lineages of this supergroup. Hence, it may be that the common ancestor of aphelids with fungi could be a free-living organism with a wide range of trophic possibilities, rather than a specialized endobiont.

(2) In this connection, it is difficult to understand whether the reduction in the actin-associated protein complex CCC in aphelids [9] is a feature originating from a common ancestor with fungi. If the disappearance/reduction in the CCC complex in fungi is associated with the loss of phagocytosis, the reasons for its partial decrease in aphelids are still unknown. This is probably due to the parasitoid feeding of aphelids. If the specific feeding habits of fungi and aphelids were formed after the separation of the lineages, the reduction in the CCC complex could occur independently, in each case for its own specific reason, although the result of the reduction seems to be similar. In fungal ancestry, this course of events is very common [47].

(3) The presence of chitin processing enzymes in aphelids and their homology with fungal ones [9], as well as the homology of the protein acting in the infection tube of aphelidian cyst with the hyphal polarization protein [6], reveals an element of cyst germination machinery of the ancestor of aphelids and fungi. However, the noted cyst feature does not indicate that it was an infectious agent and, in general, does not say how the cyst functioned in a common ancestor. The cyst with chitinous wall and chitin processing enzymes are characteristic of most unicellular opisthokonts and, obviously, represent symplesiomorphies of opisthokonts [46,48,49].

(4) Even the zoosporic life cycle, which is shared by fungi and aphelids, also seems to be a symplesiomorphy of Opisthokonta [48,49]. Moreover, some evidence allows us to raise the question: might it be formed several times in different lineages on the basis of an ancestral ability for cellular polymorphism? This question is possible due to the proposal that the genetic basis of the life cycles of fungi and aphelids may be different [50], as well as the recent discovery of basal Holozoa with a high capacity for cellular polymorphism [46], which could be inherited from the common ancestor of Opisthokonta. Such an assumption looks unexpected but will not be so surprising given the wide convergence in morphogenetic processes in fungi [47,51].

Summing up, it turns out that all similar features of fungi and aphelids are either symplesiomorphies, characteristic of all Opisthokonta supergroup, or apomorphies that can be realized in any way of life, or probable evolutionary parallelisms. The wide distribution of convergences and parallelisms in fungi, which complicates and “confuses” the analysis of their evolution, has been repeatedly noted by various authors [47,51]. About the common ancestor of aphelids with fungi, one can only say that it somehow fed on algae and had either a well-established zoospore life cycle or cellular polymorphism including an amoeba, flagellate and cyst. Within algae nutrition and polymorphic abilities, there is still insufficient data for further refinement.

It can be reasonably assumed that the aforementioned genes for the chitin processing enzyme and growth tube proteins served as a preadaptation to the appearance of the fungal trait complex. However, these cyst-associated genes must have switched to functioning at the vegetative stage, or, conversely, the cyst must have acquired vegetative abilities [8]. In any case, the appearance of morphophysiological features of fungi was obviously based on the evolution of regulatory genes that changed the timing of expression of some components in gene interaction cascades. At the same time, the prerequisites that made this switch adaptive should have been osmotrophy and extracellular digestion [45], from which the fungi have started evolving.

In the field of taxonomy, the absence of unambiguous fungal characters in Aphelida makes desirable further discussion on the composition of the taxon Fungi. What are the apomorphies of this taxon after all? Should the diagnosis of Fungi include morphological and physiological characters, or can this taxon be characterized only by a common set of genes and proteins regardless of their functions? Probably, the solution to the question of the inclusion of aphelids and other “early divergent fungal lineages” in the Fungi should be sought in two directions: (1) by studying the set of similarities and differences in fungi and closely related organisms and (2) by discussing the rules of description of the kingdom Fungi. Obviously, for these purposes, further studies on the genomes and the genetic basis of the morphogenesis of aphelids and fungi are required.

## Figures and Tables

**Table 1:** Comparison of the genome assemblies of *Aphelidium insulamus* and closely related species.

Species	Assembly Size (bp)	Num Scaffolds	Scaffold N50 (bp)	Average Scaffold (bp)	Largest Scaffold (bp)	GC, %	Num Genes	Num Proteins	Single Copy BUSCOs (%)
*Aphelidium insulamus*	18,927,283	274	252,907	69,078	1,020,338	52.05	7925	7820	91.4%
*Amoeboaphelidium protococcarum*	24,734,778	258	2,170,272	95,871	3,250,117	40.50	13,180	13,180	92.7%
*Amoeboaphelidium occidentale*	13,559,732	951	73,507	14,258	366,412	39.93	7568	7495	91.4%
*Gonapodya prolifera*	48,794,828	352	347,324	138,622	1,572,201	51.75	13,911	13,831	93.4%
*Blyttiomyces helicus*	46,468,912	8398	6675	5533	73,981	53.75	12,446	12,167	55.4%
*Powellomyces hirtus*	26,238,698	482	157,542	54,437	764,225	51.37	6536	6536	96.4%
*Spizellomyces punctatus*	24,131,112	38	1,465,700	635,029	2,242,449	47.16	9164	9422	97.4%

**Table 2:** The total numbers and accession numbers (NCBI) of SP proteins found in studied opisthokont species. Large groups are marked with colors.

						
Aphelida	Zoosporic Fungi	Dikaryan Fungi	Rozellida	Filasterea	Choanoflagellata	Metazoa
Species	Designation in the Phylogenetic Tree	Number of SP Proteins	Accession Numbers			
*Aphelidium insulamus*	aphins	3	OR609_004515-T1 (FUN_002943-T1), OR609_000014-T1 (FUN_001752-T1), OR609_006691-T1 (FUN_007167-T1)			
*Paraphelidium tribonematis*	partrib	1	Partr_v1_DN257			
*Amoeboaphelidium protococcarum*	amoebprot	8	KAI3642224.1, KAI3631285.1, KAI3647471.1, KAI3641405.1, KAI3636887.1, KAI3651751.1, KAI3653019.1, KAI3638028.1			
*Amoeboaphelidium occidentale*	amoeboccid	2	KAI3658561.1, KAI3659551.1			
*Blyttiomyces helicus*	blitt	3	RKO83577.1, RKO87217.1, RKO84726.1			
*Gonapodya prolifera*	gonap	6	KXS20085.1, KXS21772.1, KXS18310.1, KXS19020.1, KXS14312.1, KXS18846.1			
*Powellomyces hirtus*	powell	4	TPX57964, TPX61303, TPX53442, TPX57119			
*Spizellomyces punctatus*	spizel	5	XP_016610136.1, XP_016608866.1, XP_016607884.1, XP_016608771.1, XP_016607634.1			
*Synchytrium endobioticum*	synchyt	4	TPX52967.1, TPX40513.1, TPX48127.1, TPX35163.1			
*Agaricus bisporus*	agar	26	XP_006454719.1, XP_006461850.1, XP_006453951.1, XP_006456284.1, XP_006459401.1, XP_006454620.1, XP_006457533.1, XP_006460576.1, XP_006459994.1, XP_006459113.1, XP_006463002.1, XP_006459487.1, XP_006459489.1, XP_006459491.1, XP_006458691.1, XP_006459490.1, XP_006456584.1, XP_006460947.1, XP_006455907.1, XP_006455078.1, XP_006456754.1, XP_006456796.1, XP_006456892.1, XP_006463354.1, XP_006459525.1, XP_006458285.1			
*Neurospora crassa*	neurosp	34	XP_959573.2, XP_959411.2, XP_965713.1, XP_955977.1, XP_962392.2, XP_958069.2, XP_960171.3, XP_956001.1, XP_959563.1, XP_960000.1, XP_964364.3, XP_958937.1, XP_956491.3, XP_959616.1, XP_959582.3, XP_961039.2, XP_963898.1, XP_963341.3, XP_963320.3, XP_960136.3, XP_960988.3, XP_961779.1, XP_960559.2, XP_958139.1, XP_955927.2, XP_964352.1, XP_963873.1, XP_965499.1, XP_960547.2, XP_963122.1, XP_957424.1, XP_001728155.2, XP_963801.1, XP_957394.1			
*Saccharomyces cerevisiae*	sacch	30	NP_010087.1, NP_010143.1, NP_011960.2, NP_116644.1, NP_013724.1, NP_013182.1, NP_011962.1, NP_010632.1, NP_012316.1, NP_010629.3, NP_010630.1, NP_011964.1, NP_014486.1, NP_012321.1, NP_014470.1, NP_010845.1, NP_010036.1, NP_012692.3, NP_014081.1, NP_010825.3, NP_010785.1, NP_014538.2, NP_012694.1, NP_010034.1, NP_011805.3, NP_116613.1, NP_009857.1, NP_010675.3, NP_009800.1, NP_011411.3			
*Rozella allomycis*	roz	2	EPZ36062, EPZ31040			
*Capsaspora owczarzaki*	capsa	1	XP_004342757.1			
*Monosiga brevicollis*	monos	5	XP_001748408.1, XP_001745016.1, XP_001745420.1, XP_001748510.1, XP_001744797.1			
*Salpingoeca rosetta*	salpin	5	XP_004992344.1, XP_004991932.1, XP_004990085.1, XP_004994833.1, XP_004991367.1			
*Amphimedon queenslandica*	amphim	13	XP_019857806.1, XP_019857805.1, XP_019851521.1, XP_011406421.1, XP_003389392.2, XP_003383220.1, XP_019863478.1, XP_011402949.1, XP_019856859.1, XP_003384062.3, XP_003385376.1, XP_011408594.2, XP_003384855.1			

**Table 3:** Fifteen first hits of BLAST search for, *A. protococcarum* protein KAI3651751.1 as a query in general nucleotide database (nt). Blue letters—Aphelida, green letters—Amoebozoa, russet letters—Bacteria.

Description	Max Score	Total Score	Query Cover	E Value	Per. Ident	Acc. Len	Accession
Hypothetical protein MP228_003054 (*Amoeboaphelidium protococcarum*)	1009	1009	100%	0.0	100.00%	501	KAI3651751.1
Hypothetical protein MIR68_003639 (*Amoeboaphelidium protococcarum*)	882	882	100%	0.0	92.66%	503	KAI3638028.1
Hypothetical protein MP228_002444 (*Amoeboaphelidium protococcarum*)	881	881	100%	0.0	92.64%	502	KAI3653019.1
Hypothetical protein MIR68_005154 (*Amoeboaphelidium protococcarum*)	863	863	100%	0.0	90.89%	504	KAI3636887.1
Hypothetical protein MP638_005237 (*Amoeboaphelidium occidentale*)	196	196	89%	2 × 10^52^	30.95%	483	KAI3659551.1
Sugar transporter family protein (*Tieghemostelium lacteum*)	197	197	93%	7 × 10^52^	30.42%	631	KYQ90579.1
Sugar transporter family protein (*Dictyostelium discoideum* AX4)	190	190	92%	4 × 10^49^	30.29%	630	XP_642246.1
Sugar porter family MFS transporter (*Bacteroidales bacterium*)	186	186	92%	1 × 10^48^	28.48%	495	MBS3771599.1
Sugar porter family MFS transporter (*Bacteroidales bacterium*)	185	185	92%	2 × 10^48^	28.14%	495	MBS3775699.1
Sugar porter family MFS transporter (*Acetilactobacillus jinshanensis*)	181	181	90%	6 × 10^47^	31.30%	467	WP_133441301.1
Sugar porter family MFS transporter (*Bacteroidota bacterium*)	181	181	92%	6 × 10^47^	27.35%	486	NBC83568.1
Sugar porter family MFS transporter (uncultured bacterium)	181	181	90%	7 × 10^47^	31.30%	467	URL60617.1
Sugar porter family MFS transporter (*Lentilactobacillus* sp. SPB1-3)	178	178	90%	5 × 10^46^	29.00%	467	WP_268912157.1
Sugar porter family MFS transporter (*Lentilactobacillus curieae*)	177	177	90%	6 × 46	29.93%	460	WP_035166644.1
Sugar porter family MFS transporter (*Secundilactobacillus hailunensis*)	176	176	90%	4 × 45	30.79%	463	WP_137631226.1

## Data Availability

https://www.ncbi.nlm.nih.gov/bioproject/?term=PRJNA902644.

## Supplementary Materials

The following supporting information can be downloaded at: https://www.mdpi.com/article/10.3390/jof9101021/s1, Supplementary Table S1: Occurrence of PFAM domains in genomes; Supplementary Tree S2: The phylogenetic tree of SP proteins of the studied opisthokont species. Full version; Supplementary Tree S3: The phylogenetic tree of SP proteins of the studied fungal, aphelid and bacterial species. Full version.

## References

[1] Gromov B.V. (2000). Algal parasites of the genera Aphelidium, Amoeboaphelidium and Pseudoaphelidium from the Cienkovski’s “Monadea” group as representatives of new class. Zool. Z..

[2] Karpov S.A., Mamkaeva M.A., Aleoshin V.V., Nassonova E., Lilje O., Gleason F.H. (2014). Morphology, phylogeny, and ecology of the aphelids (Aphelidea, Opisthokonta) and proposal for the new superphylum Opisthosporidia. Front. Microbiol..

[3] Letcher P.M., Lopez S., Schmieder R., Lee P.A., Behnke C., Powell M.J., McBride R.C. (2013). Characterization of *Amoeboaphelidium protococcarum*, an algal parasite new to the cryptomycota isolated from an outdoor algal pond used for the production of biofuel. PLoS ONE.

[4] Adl S.M., Bass D., Lane C.E., Lukes J., Schoch C.L., Smirnov A., Agatha S., Berney C., Brown M.W., Burki F. (2019). Revisions to the classification, nomenclature, and diversity of eukaryotes. J. Eukaryot. Microbiol..

[5] Letcher P.M., Powell M.J. (2019). A taxonomic summary of *Aphelidiaceae*. IMA Fungus.

[6] Torruella G., Grau-Bové X., Moreira D., Karpov S.A., Burns J.A., Sebé-Pedrós A., Völcker E., López-García P. (2018). Global transcriptome analysis of the aphelid *Paraphelidium tribonemae* supports the phagotrophic origin of fungi. Commun. Biol..

[7] Tedersoo L., Sánchez-Ramírez S., Kõljalg U., Bahram M., Döring M., Schigel D., May T., Ryberg M., Abarenkov K. (2018). High-level classification of the Fungi and a tool for evolutionary ecological analyses. Fungal Divers..

[8] Galindo L.J., Torruella G., López-García P., Ciobanu M., Gutiérrez-Preciado A., Karpov S.A., Moreira D. (2022). Phylogenomics supports the monophyly of aphelids and fungi and identifies new molecular synapomorphies. Syst. Biol..

[9] Mikhailov K.V., Karpov S.A., Letcher P.M., Lee P.A., Logacheva M.D., Penin A.A., Nesterenko M.A., Pozdnyakov I.R., Potapenko E.V., Sherbakov D.Y. (2022). Genomic analysis reveals cryptic diversity in aphelids and sheds light on the emergence of Fungi. Curr. Biol..

[10] Guarro J., Gené J., Stchigel A.M. (1999). Developments in fungal taxonomy. Clin. Microbiol. Rev..

[11] Margulis L., Chapman M.J. (2009). Chapter Four—Kingdom Fungi. Kingdoms and Domains.

[12] Naranjo-Ortiz M.A., Gabaldón T. (2019). Fungal evolution: Diversity, taxonomy and phylogeny of the Fungi. Biol. Rev..

[13] Richards T.A., Leonard G., Wideman J.G. (2017). What defines the “Kingdom” Fungi?. Microbiol. Spectr..

[14] Henderson P.J. (1990). The homologous glucose transport proteins of prokaryotes and eukaryotes. Res. Microbiol..

[15] Pao S.S., Paulsen I.T., Saier M.H. (1998). Major facilitator superfamily. Microbiol. Mol. Biol. Rev..

[16] Yan N. (2013). Structural advances for the major facilitator superfamily (MFS) transporters. Trends Biochem. Sci..

[17] InterPro—Classification of Protein Families Major Facilitator Superfamily Domain. https://www.ebi.ac.uk/interpro/entry/InterPro/IPR020846/.

[18] Reddy V.S., Shlykov M.A., Castillo R., Sun E.I., Saier M.H. (2012). The major facilitator superfamily (MFS) revisited. FEBS J..

[19] Gonçalves C., Coelho M.A., Salema-Oom M., Gonçalves P. (2016). Stepwise functional evolution in a fungal sugar transporter family. Mol. Biol. Evol..

[20] Merényi Z., Krizsán K., Sahu N., Liu X., Bálint B., Stajich J., Spatafora J.W., Nagy L.G. (2022). Taxonomic vs genomic fungi: Contrasting evolutionary loss of protistan genomic heritage and emergence of fungal novelties. bioRxiv.

[21] Van Dijck P., Brown N.A., Goldman G.H., Rutherford J., Xue C., Van Zeebroeck G. (2017). Nutrient sensing at the plasma membrane of fungal cells. Microbiol. Spectr..

[22] Bisson L.F., Coons D.M., Kruckeberg A.L., Lewis D.A. (1993). Yeast sugar transporters. Crit. Rev. Biochem. Mol. Biol..

[23] Goffeau A., Park J., Paulsen I.T., Jonniaux J.L., Dinh T., Mordant P., Saier M.H. (1997). Multidrug-resistant transport proteins in yeast: Complete inventory and phylogenetic characterization of yeast open reading frames with the major facilitator superfamily. Yeast.

[24] Dias P.J., Sá-Correia I. (2013). The drug:H^+^ antiporters of family 2 (DHA2), siderophore transporters (ARN) and glutathione:H+antiporters (GEX) have a common evolutionary origin in hemiascomycete yeasts. BMC Genom..

[25] Karpov S.A., Vishnyakov A.E., López-García P., Zorina N.A., Ciobanu M., Tcvetkova V.S., Moreira D. (2020). Morphology and molecular phylogeny of *Aphelidium insulamus* sp. nov. (Aphelida, Opisthosporidia). Protistology.

[26] Fahrni J.F., Bolivar I., Berney C., Nassonova E., Smirnov A., Pawlowski J. (2003). Phylogeny of lobose amoebae based on actin and small-subunit ribosomal RNA genes. Mol. Biol. Evol..

[27] Kolmogorov M., Yuan J., Lin Y., Pevzner P. (2019). Assembly of long error-prone reads using repeat graphs. Nat. Biotechnol..

[28] Challis R., Richards E., Rajan J., Cochrane G., Blaxter M. (2020). BlobToolKit–interactive quality assessment of genome assemblies. G3-Genes Genom. Genet..

[29] Li H. (2018). Minimap2: Pairwise alignment for nucleotide sequences. Bioinformatics.

[30] Vaser R., Sović I., Nagarajan N., Šikić M. (2017). Fast and accurate de novo genome assembly from long uncorrected reads. Genome Res..

[31] Walker B.J., Abeel T., Shea T., Priest M., Abouelliel A., Sakthikumar S., Cuomo C.A., Zeng Q., Wortman J., Young S.K. (2014). Pilon: An integrated tool for comprehensive microbial variant detection and genome assembly improvement. PLoS ONE.

[32] Simão F.A., Waterhouse R.M., Ioannidis P., Kriventseva E.V., Zdobnov E.M. (2015). BUSCO: Assessing genome assembly and annotation completeness with single-copy orthologs. Bioinformatics.

[33] Gurevich A., Saveliev V., Vyahhi N., Tesler G. (2013). QUAST: Quality assessment tool for genome assemblies. Bioinformatics.

[34] Palmer J., Stajich J. (2020). Funannotate v1.8.1: Eukaryotic Genome Annotation (v1.8.1). Zenodo.

[35] Haas B.J., Salzberg S.L., Zhu W., Pertea M., Allen J.E., Orvis J., White O., Buell C.R., Wortman J.R. (2008). Automated eukaryotic gene structure annotation using EVidenceModeler and the Program to Assemble Spliced Alignments. Genome Biol..

[36] Käll L., Krogh A., Sonnhammer E.L. (2007). Advantages of combined transmembrane topology and signal peptide prediction—The Phobius web server. Nucleic Acids Res..

[37] Chan P.P., Lowe T.M., Kollmar M. (2019). tRNAscan-SE: Searching for tRNA genes in genomic sequences. Gene Prediction. Methods in Molecular Biology.

[38] Jones P., Binns D., Chang H.-Y., Fraser M., Li W., McAnulla C., McWilliam H., Maslen J., Mitchell A., Nuka G. (2014). InterProScan 5: Genome-scale protein function classification. Bioinformatics.

[39] Camacho C., Coulouris G., Avagyan V., Ma N., Papadopoulos J., Bealer K., Madden T.L. (2009). BLAST+: Architecture and applications. BMC Bioinform..

[40] Wheeler T.J., Eddy S.R. (2013). nhmmer: DNA homology search with profile HMMs. Bioinformatics.

[41] Di Tommaso P., Moretti S., Xenarios I., Orobitg M., Montanyola A., Chang J.M., Taly J.F., Notredame C. (2011). T-Coffee: A web server for the multiple sequence alignment of protein and RNA sequences using structural information and homology extension. Nucleic Acids Res..

[42] Capella-Gutierrez S., Silla-Martinez J.M., Gabaldon T. (2009). trimAl: A tool for automated alignment trimming in large-scale phylogenetic analyses. Bioinformatics.

[43] Nguyen L.T., Schmidt H.A., von Haeseler A., Minh B.Q. (2015). IQ-TREE: A fast and effective stochastic algorithm for estimating maximum-likelihood phylogenies. Mol. Biol. Evol..

[44] Ocaña-Pallarès E., Williams T.A., López-Escardó D., Arroyo A.S., Pathmanathan J.S., Bapteste E., Tikhonenkov D.V., Keeling P.J., Szöllősi G.J., Ruiz-Trillo I. (2022). Divergent genomic trajectories predate the origin of animals and fungi. Nature.

[45] Chang Y., Wang S., Sekimoto S., Aerts A.L., Choi C., Clum A., LaButti K.M., Lindquist E.A., Ngan C.Y., Ohm R.A. (2015). Phylogenomic analyses indicate that early fungi evolved digesting cell walls of algal ancestors of land plants. Genome Biol. Evol..

[46] Tikhonenkov D.V., Hehenberger E., Esaulov A.S., Belyakova O.I., Mazei Y.A., Mylnikov A.P., Keeling P.J. (2020). Insights into the origin of metazoan multicellularity from predatory unicellular relatives of animals. BMC Biol..

[47] Brun S., Silar P., Pontarotti P. (2010). Convergent evolution of morphogenetic processes in Fungi. Evolutionary Biology—Concepts, Molecular and Morphological Evolution.

[48] Kożyczkowska A., Najle S.R., Ocaña-Pallarès E., Aresté C., Shabardina V., Ara P.S., Ruiz-Trillo I., Casacuberta E. (2021). Stable transfection in protist *Corallochytrium limacisporum* identifies novel cellular features among unicellular animals relatives. Curr. Biol..

[49] Torruella G., de Mendoza A., Grau-Bové X., Antó M., Chaplin M.A., del Campo J., Eme L., Pérez-Cordón G., Whipps C.M., Nichols K.M. (2015). Phylogenomics reveals convergent evolution of lifestyles in close relatives of animals and fungi. Curr. Biol..

[50] Pozdnyakov I.R., Zolotarev A.V., Karpov S.A. (2021). Comparative analysis of zoosporogenesis’ genes of the bastoclad *Blastocladiella emersonii* and the aphelid *Paraphelidium tribonematis* reveals the new directions of evolutionary research. Protistology.

[51] Nagy L.G., Kovács G.M., Krizsán K. (2018). Complex multicellularity in fungi: Evolutionary convergence, single origin, or both?. Biol. Rev. Camb. Philos. Soc..

